# Norovirus P Particle Efficiently Elicits Innate, Humoral and Cellular Immunity

**DOI:** 10.1371/journal.pone.0063269

**Published:** 2013-04-29

**Authors:** Hao Fang, Ming Tan, Ming Xia, Leyi Wang, Xi Jiang

**Affiliations:** 1 Division of Infectious Diseases, Cincinnati Children's Hospital Medical Center, Cincinnati, Ohio, United States of America; 2 Department of Pediatrics, University of Cincinnati College of Medicine, Cincinnati, Ohio, United States of America; Tulane University, United States of America

## Abstract

Norovirus (NoV) P domain complexes, the 24 mer P particles and the P dimers, induced effective humoral immunity, but their role in the cellular immune responses remained unclear. We reported here a study on cellular immune responses of the two P domain complexes in comparison with the virus-like particle (VLP) of a GII.4 NoV (VA387) in mice. The P domain complexes induced significant central memory CD4^+^ T cell phenotypes (CD4^+^ CD44^+^ CD62L^+^ CCR7^+^) and activated polyclonal CD4^+^ T cells as shown by production of Interleukin (IL)-2, Interferon (IFN)-γ, and Tumor Necrosis Factor (TNF)-α. Most importantly, VA387-specific CD4^+^ T cell epitope induced a production of IFN-γ, indicating an antigen-specific CD4^+^ T cell response in P domain complex-immunized mice. Furthermore, P domain complexes efficiently induced bone marrow-derived dendritic cell (BMDC) maturation, evidenced by up-regulation of co-stimulatory and MHC class II molecules, as well as production of IL-12 and IL-1β. Finally, P domain complex-induced mature dendritic cells (DCs) elicited proliferation of specific CD4^+^ T cells targeting VA387 P domain. Overall, we conclude that the NoV P domain complexes are efficiently presented by DCs to elicit not only humoral but also cellular immune responses against NoVs. Since the P particle is highly effective for both humoral and cellular immune responses and easily produced in *Escherichia coli (E. coli)*, it is a good choice of vaccine against NoVs and a vaccine platform against other diseases.

## Introduction

Noroviruses (NoVs) are important pathogens causing epidemics of acute gastroenteritis in humans [1]. They are a group of single-stranded, positive sense RNA viruses constituting the *Norovirus* genus in the family *Caliciviridae*. Structurally NoVs are nonenveloped viruses that contain an outer protein capsid encapsulating an RNA genome. The NoV genome is ∼7.5 kb in length comprising three open reading frames (ORFs). ORF1 encodes six non-structural proteins, while ORF2 and 3 encode the major (VP1) and minor (VP2) structural proteins, respectively [2]. Due to the lack of an efficient cell culture system and a small animal model, our understanding on pathogenesis, immunology and replication of NoVs remains limited.

**Figure 1:**
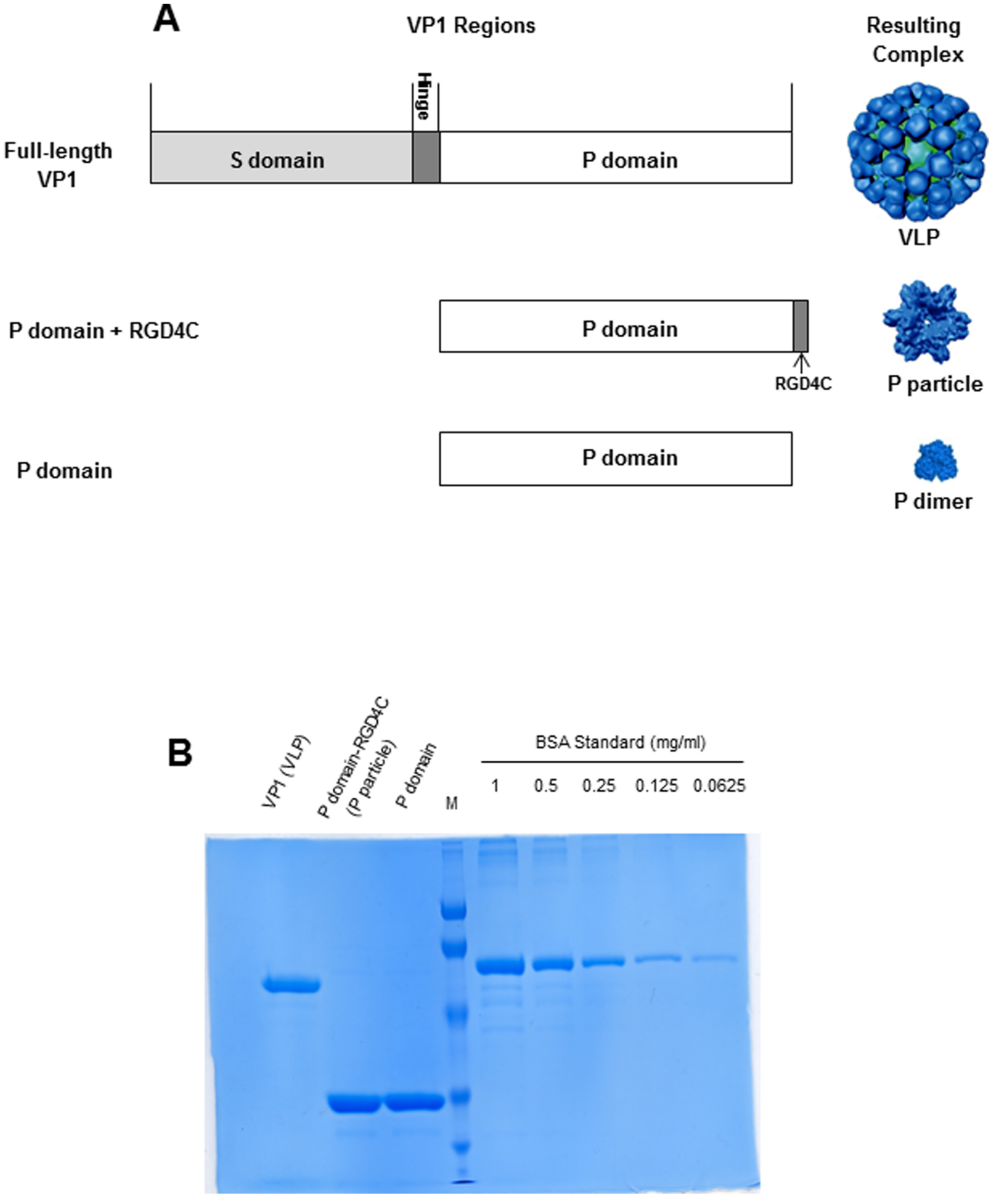
Norovirus P domain complexes used in this study. (A) Schematic illustration of Norovirus VP1, P domain, and their corresponding complex formation. VP1 is composed of an N-terminal S and a C-terminal P domain that are linked by a short hinge. Full-length VP1 forms virus-like particle (VLP), while the modified P domain with an RGD4C peptide and the unmodified P domain form the P particles and P dimers, respectively. RGD4C is a cysteine-containing peptide with sequences of CDCRGDCFC. (B) Protein quality and their concentration determination. VP1 (VLP) and two P domains were analyzed by SDS PAGE along with the bovine serum albumin (BSA) at different amounts to determine the concentration of VP1 and P proteins.

*In vitro* expression of NoV VP1 spontaneously assembles into virus-like particles (VLPs) that are morphologically and antigenically indistinguishable from the authentic virus (Figure 1A). NoV VLP has been exploited as a surrogate and a vaccine candidate of NoV owing to the fact that NoVs are not cultivatable in the laboratory. NoV VP1 can be divided into two major domains, the shell (S) and the protruding (P) domains [3]. While the S domain is responsible for building the interior shell, the P domain forms the exterior protrusions of the virus, forming the major antigenic structures of NoVs. The S and the P domains may be structurally and functionally independent. Expression of the S domain forms the small, thin-layer S particle [4], [5] without binding function to histo-blood group antigens (HBGAs), the attachment factors or receptors of NoVs [6]–[10]. In contrast, production of the P domain alone assembles different P domain complexes, including the P dimer [5], [11]–[14], the 12mer small P particle [15], the 24mer P particle [9], [16], [17] (Figure 1). These P domain complexes are interchangeable under certain conditions [18] and all P domain complexes retain binding function to HBGAs [9], [16], [19], indicating that the P domain is the carbohydrate binding domain [11]–[14]. A truncated P domain protein without the C-terminal arginine-cluster, named P polypeptide, was found in large amount in stools of NoV infected patients [20]–[22], though its biological significance remains unknown.

Currently there are no vaccine and antivirals available for NoVs. Because human NoVs are uncultivable, conventional vaccines such as the live attenuated or inactivated vaccines are impossible for NoVs. Thus, subunit NoV vaccines, such as VLP- and P particle-based vaccines, are under development [17], [23]–[28]. A Norwalk virus (GI.1)-based VLP vaccine has been in the phase II human trial, which showed safe and protection against infection and gastroenteritis caused by challenge of the homologous Norwalk virus [23], [29]. Due to the lack of a small animal model for human NoVs, mice and rabbits were used to evaluate the immunogenicity of NoV vaccine candidates [25], [27], [30]. In addition, the P particle has been developed into a vaccine platform for antigen presentation for enhanced immunity against foreign antigens for vaccine development [17], [27], [28], [30], [31]. Thus, a study to understand the mechanisms of host immune responses, particularly the cellular immune responses, to NoV VLP and different P domain complexes is necessary. Because the P domain represents only half of the viral capsid protein, we performed a direct comparison of cellular immunity of mice to two P domain complexes (the P particle and P dimer) with that to the full-length capsid VLPs. Our data revealed that while both P domain complexes are efficiently presented by dendritic cells and elicited cellular immunity, the P particle induced high humoral and cellular immune responses similar to those induced by VLP. These findings supported the notion of the P particle as a promising vaccine against NoVs and a useful vaccine platform for dual vaccine development against other diseases.

## Materials and Methods

### Reagents

Various reagents were purchased from following companies: mouse monoclonal antibodies anti-CD3 (145-2C11), anti-CD4 (GK1.5), anti-CD8 (53–6.7), anti-CD44 (IM7), anti-CD62L (MEL-14), anti-CCR7 (4B12), anti-CD11c (N418), anti-CD40 (3/23), anti-CD80 (16-10A1), anti-CD86 (GL-1), anti-I-A^b^ (AF6-120.1), anti-IL-2 (JES6-5H4), anti-IFN-γ (XMG1.2), anti-TNF-α (MP6-XT22) from Biolegend (San Diego, CA), purified anti-mouse IL-12 p70 (C18.2) and biotin anti-mouse IL-12/IL-23 p40 (C17.8) from Biolegend, purified anti-mouse IFN-γ (R4-6A2) and biotin anti-mouse IFN-γ (XMG1.2) from Biolegend, ELISA set of murine IL-1β from BD Bioscience (San Jose, CA), 10x RBC lysis buffer and fixation/permeabilization buffers from eBioscience (San Diego, CA), Alexa Fluor 488® protein labeling kit from Invitrogen (Grand Island, NY), HRP-conjugated goat anti-mouse IgG, IgG1 and rabbit anti-mouse IgG2a, 2b from MP Biomedicals (Solon, OH, USA), and Polymyxin B from Sigma-Aldrich (St. Louis, MO). CD4^+^ T cell epitope (FYQEAAPAQSDVAL) targeting NoV GII.4 strain VA387 were predicted based on T cell epitope prediction tools from *Immune Epitope Database (IEDB) Analysis Resource* (website: http://tools.immuneepitope.org/main/) and synthesized by GenScript (Piscataway, NJ).

### Expression and purification of recombinant P proteins

Baculovirus-expressed VLP of NoV strain VA387 (GII.4) was obtained from LigoCyte Pharmaceuticals. P dimers and P particles were expressed in *E. coli* (BL21, DE3) with an induction of 0.25 mM isopropyl-β-D-thiogalactopyranoside (IPTG) at room temperature (∼23°C) overnight as described elsewhere [5], [9], [16], [20]. Purification of the glutathione S-transferase (GST)-P domain fusion proteins were performed using resin of Glutathione Sepharose 4 Fast Flow (GE Healthcare Life Sciences, Piscataway, NJ) according to the manufacturer's instruction. GST was removed from the target proteins by thrombin (GE Healthcare Life Sciences) cleavage on beads 1x phosphate buffer saline (PBS, pH 7.4). The P domain complexes were further purified by Gel filtration chromatography that was carried out through an AKTA FPLC System (GE Healthcare Life Sciences) as described previously [9].

### Vaccination

Specific pathogen-free female BALB/c mice were purchased from Harlan-Sprague-Dawley (Indianapolis, IN) and immunized at four weeks of age. The animals were housed in a temperature-controlled environment with 12 h light/dark cycles, and received food and water under the control of Veterinary Services of CCHMC (Cincinnati Children Hospital Medical Center). A dose of 30 µg (at concentration of 1 µg/µl) of purified VA387 VLP, P particle, or P dimer were administered to each mouse (n = 5 mice per group) intranasally without adjuvant after inhalational anesthesia of isoflurane. Mice received three doses of immunization with a two-week interval. For comparison of immune responses, PBS was inoculated to mice (n = 5 mice per group) intranasally as a negative control. Sera were collected from each mouse prior to the first immunization and one week after the final immunization.

### Determination of antibody titers in sera

Antibody titer in serum from individual mouse is measured by an enzyme-linked immunosorbent assay (ELISA) using purified P dimer, as coating antigen [27]. Sera were serially diluted in 2 folds starting at 1∶100 and the specific antibody titers were defined as the endpoint dilution with an OD_450_ equal or larger than 0.2.

### Flow Cytometry for surface molecule staining and intracellular cytokine staining

One week after the last immunization, spleens were collected from mice. Suspension of splenocytes was prepared by lysing red blood cells with 1xRBC lysis buffer (eBiosciences). Cells were washed with FACS buffer (1xPBS with 1% FBS and 0.01% NaN_3_) and then stained with fluorochrome-conjugated antibodies at 4°C. For phenotypic analysis of CD4^+^ T cells, CD4, CD44, CD62L and CCR7 were used as makers for central memory phenotype [32], [33].

For intracellular cytokine staining, splenocytes were resuspended in RPMI-1640 with 3 µg/ml of brefeldin A and 5 µg/ml of VA387 CD4^+^ T cell epitope for 6 hours. PMA (25 ng/ml) and ionomycin (500 ng/ml) were used as positive control for cytokine production. After stimulation, cells were washed with FACS buffer and stained with surface makers such as CD3 and CD4 for 15 minutes at 4°C. Then cells were washed twice and fixed with Fixation Buffer overnight at 4°C (eBiosciences). Next day, cells were washed with 1x permeabilization buffer twice and stained with fluorochrome-conjugated cytokine antibodies for 30 minutes at 4°C. Cells were washed twice with 1x permeabilization buffer and twice with FACS buffer, and then resuspended in FACS buffer for acquisition. All samples were acquired by BD FACSCanto, BD LSR II or BD Accuri™ C6 and data was analyzed by FlowJo or Accuri™ C6 software.

### Generation and maturation of murine bone marrow-derived dendritic cells (BMDCs)

Bone marrow-derived DCs were generated as previously described [34], [35]. Briefly, mice were sacrificed and the femurs and tibias were dissected and flushed with ice-cold PBS to extrude the bone marrow. A cell strainer was used to dissociate the bone marrow to get bone marrow cell suspensions. Then, erythrocytes were lysed using 1xRBC lysis buffer, and washed cells were suspended in RPMI 1640 medium supplemented with 10% heat-inactivated fetal calf serum, penicillin (50 U/ml), streptomycin (50 μg/ml), L-glutamine (2 mM), β-mercaptoethanol (50 μM), sodium pyruvate (1 mM), sodium bicarbonate (1.5 mg/ml), and HEPES (25 mM). The bone marrow cells were cultured in 6-well plates at a density of 1×10^6^ cells/ml and incubated at 37°C in a humidified 5% CO_2_ environment. Recombinant granulocyte-macrophage colony-stimulating factor (GM-CSF) (Minneapolis, MN) was added to the cultures at a final concentration of 20 ng/ml. Culture medium and GM-CSF were replaced on days 2 and 4. Fresh culture medium with GM-CSF was replaced on day 6, and cells were harvested on day 7. This protocol routinely yielded >90% CD11c^+^ cells as determined by flow cytometry. DC maturation was analyzed as described previously [36]. In brief, BMDCs were stimulated with 10 µg/ml of NoV VLP, P particle, and P dimer for 48 hours, respectively. Cells were harvested at 24 or 48 hours and stained with fluorochrome-conjugated CD11c together with CD40, CD80, CD86 and MHC-II for either flow cytometry or ImageStreamX analysis.

### Alexa Fluor 488® labeling of VA387 P particle

This was performed using the Alexa Fluor 488® labeling kit (Invitrogen) according to the manufacturer's instruction. Fifty µl of 1 M bicarbonate were added to 0.5 ml of the 1 mg/ml P particle solution. The solution was then transferred to the vial of reactive dye and the mixture was stirred for 1 hour at room temperature. The Alexa Fluro 488® (AF488)-labeled protein was collected by passing through the purification resin. The concentration and degree of labeling were measured by the absorbance of the conjugate solution at 280 nm and 494 nm. The protein concentration was calculated as following formula: protein concentration (M)  =  [A_280_−(A_494_ ×0.11)] × dilution factor/203,000. The degree of labeling was calculated as: moles dye per mole protein  =  A_494_ × dilution factor/71,000× protein concentration (M).

### CFSE-labeled CD4^+^ T cell proliferation assay

Splenocytes were isolated from immunized mice, washed with serum-free medium for three times and then labeled with carboxyfluorescein succinimidyl ester (CFSE, Invitrogen) for 15 minutes at room temperature. At the same time, mature BMDCs pulsed with related antigen of NoV VLP, P particle or P dimer were collected as previously described. Then, CFSE-labeled splenocytes were co-incubated with antigen-induced mature BMDCs for 5 days. Finally, cells were collected, washed with FACS buffer and stained with fluorochrome-conjugated CD3 and CD4. Cells were acquired by BD FACSCanto, BD LSR II or BD Accuri™ C6 and flow data was analyzed by FlowJo or Accuri™ C6 software.

### Graphics and Statistical analysis

Graphics were made using Microsoft Office Excel 2010 and *P* values were determined by ANOVA or Chi-square test among data groups by GraphPad Prism 5 for windows (GraphPad Software, San Diego, CA).

### Ethics Statement

This study was carried out in accordance with the recommendations in the *Guide for the Care and Use of Laboratory Animals* of the National Institutes of Health. The protocols were approved by the Institutional Animal Care and Use Committee (IACUC) of the Cincinnati Children's Hospital Research Foundation (Animal Welfare Assurance Number A3108-01).

## Results

### P domain complexes induced robust antibody responses

**Figure 2:**
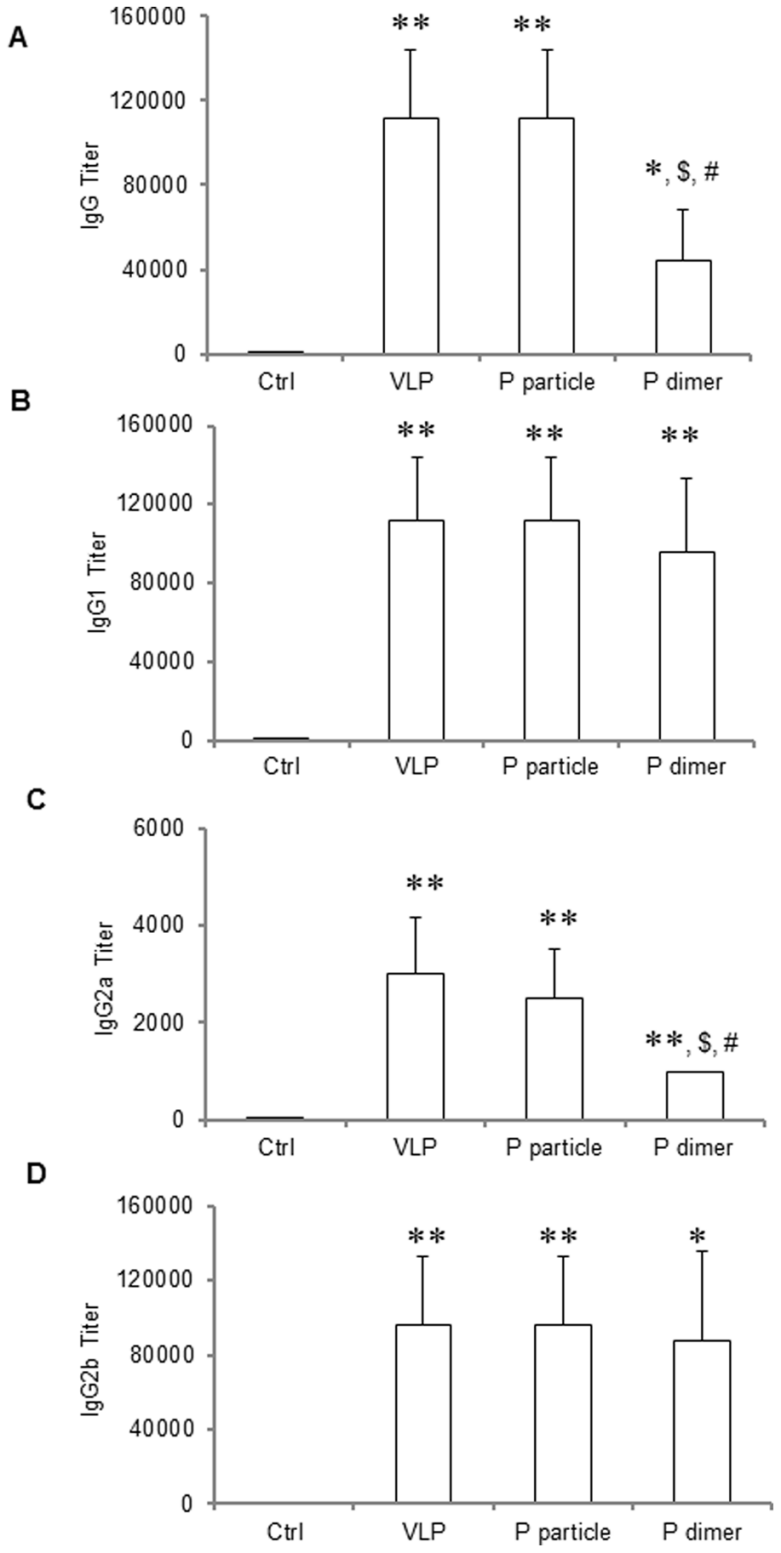
Antibodies titers in mice after immunization with NoV VLPs, P particles, and P dimers. VA387 VLPs, P particles, P dimers were administrated to the mice (30 µg/mouse, n = 5) intranasally without adjuvant for three times in a two-week interval. Mice administered with PBS were regarded as negative controls (Ctrl). Sera were collected from each mouse prior to the first and one week after the final immunization. The titers of NoV-specific total IgG (A) and its subtypes IgG1 (B), IgG2a (C) and IgG2b (D) were determined by ELISA against purified P dimer. The antibody titers were defined as the endpoint dilution with a cut off signal intensity of 0.2. ∗ *P*<0.05 and ∗∗ *P*<0.01 were calculated vs. naïve mice control (Ctrl), while $ *P*<0.05 and $$ *P*<0.01 were calculated vs. VLP and # *P*<0.05 vs. P particle, respectively. Experiments were repeated three times.

High titers of NoV specific IgG were detected in mice (n = 5 mice/group) following intranasal immunization (30 µg/mouse) of VA387 P particles and VLPs, while the IgG titer induced by the P dimer was significantly lower (Figure 2A). Similar levels of subtypes IgG 1 and IgG 2b were also detected (Figure 2B to 2D), although the P dimer induced relative lower IgG 2a titer compared with those induced by VLP or P particle.

### P domain complexes induced memory CD4^+^ T cell responses

**Figure 3:**
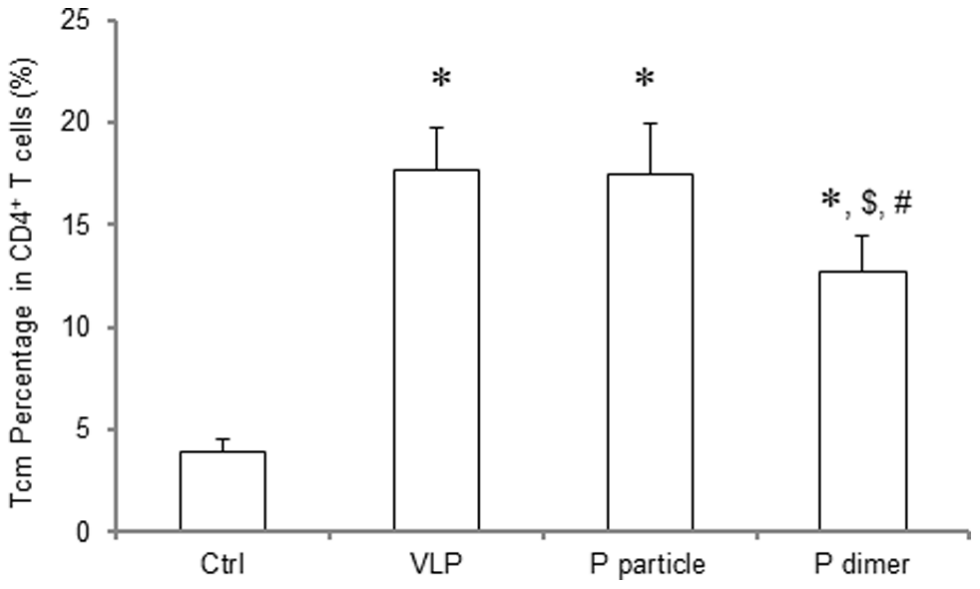
Phenotypic analysis of central memory CD4^+^ T (Tcm) cells. One week after the third immunization, spleens were collected from the VLP-, P particle- or P dimer-immunized mice. Suspension of splenocytes was prepared and the cells were stained with fluorochrome-conjugated antibodies. CD4, CD44, CD62L and CCR7 were used as makers for Tcm phenotypes [32], [33]. ∗ *P*<0.001 was calculated vs. naïve mice control (Ctrl), while $ *P*<0.05 was calculated vs. VLP and # *P*<0.05 vs. P particle, respectively. Experiments were repeated three times.

The P domain complexes also induced CD4^+^ central memory (Tcm) response in the immunized mice as shown by detection of CD4^+^CD44^+^CD62L^+^CCR7^+^ cells (Figure 3), in which the P particle induced comparable percentage of Tcm cells to that induced by VLP (*P*>0.05), while P dimer induced a lower percentage of Tcm cells (*P*<0.05 in comparison with both VLP and P particle). These differences in Tcm cell percentages may represent the potential protective effects induced by these different immunogens.

### P domain complexes induced production of IL-2, IFN-γ and TNF-α

**Figure 4:**
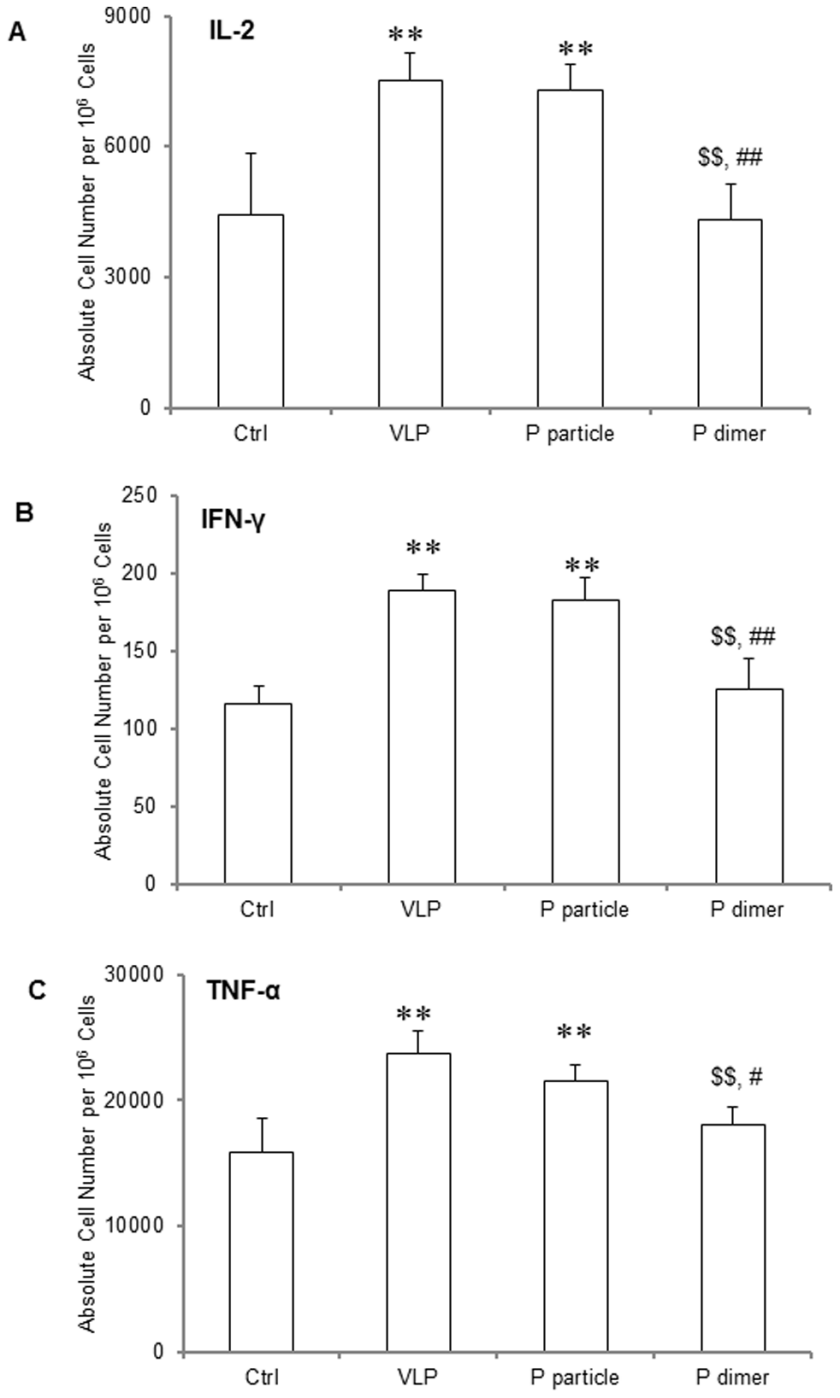
Cytokine production of IL-2, IFN-γ and TNF-α by CD4^+^ T cells in immunized mice. One week after the third immunization, spleens were collected from mice. Splenocytes were suspended in RPMI-1640 with 3 µg/ml of brefeldin A and were stimulated with PMA and ionomycin for 6 hours. The cells were then stained with surface makers CD3 and CD4, fixed with Fixation Buffer, and then stained with APC-conjugated anti-IL-2 (A), anti-IFN-γ (B) and anti-TNF-α (C) antibodies. ∗ *P*<0.05 and ∗∗ *P*<0.01 were calculated vs. naïve mice control (Ctrl), while $ *P*<0.05 and $$ *P*<0.01 were calculated vs. VLP and # *P*<0.05 and ## *P*<0.01 vs. P particle, respectively. Experiments were repeated three times.

Following stimulation with Phorbol 12-Myristate 13-Acetate (PMA) and ionomycin, significant higher IL-2 production was observed in both P particle- and VLP-immunized mice compared with naïve control group (Figure 4A, *P*<0.01), while P dimer did not induce increased IL-2 production compared with naïve control (Figure 4A, *P*>0.05), which is significantly lower than that induced by VLP and P particle (Figure 4A, *P*<0.01). No statistically significant difference was seen between VLP and P particle groups (Figure 4A, *P*>0.05). Similar results were noted in the IFN-γ and TNF-α production (Figure 4B and Figure 4C, respectively).

### P domain complexes induced NoV-specific CD4^+^ T cell responses

**Figure 5:**
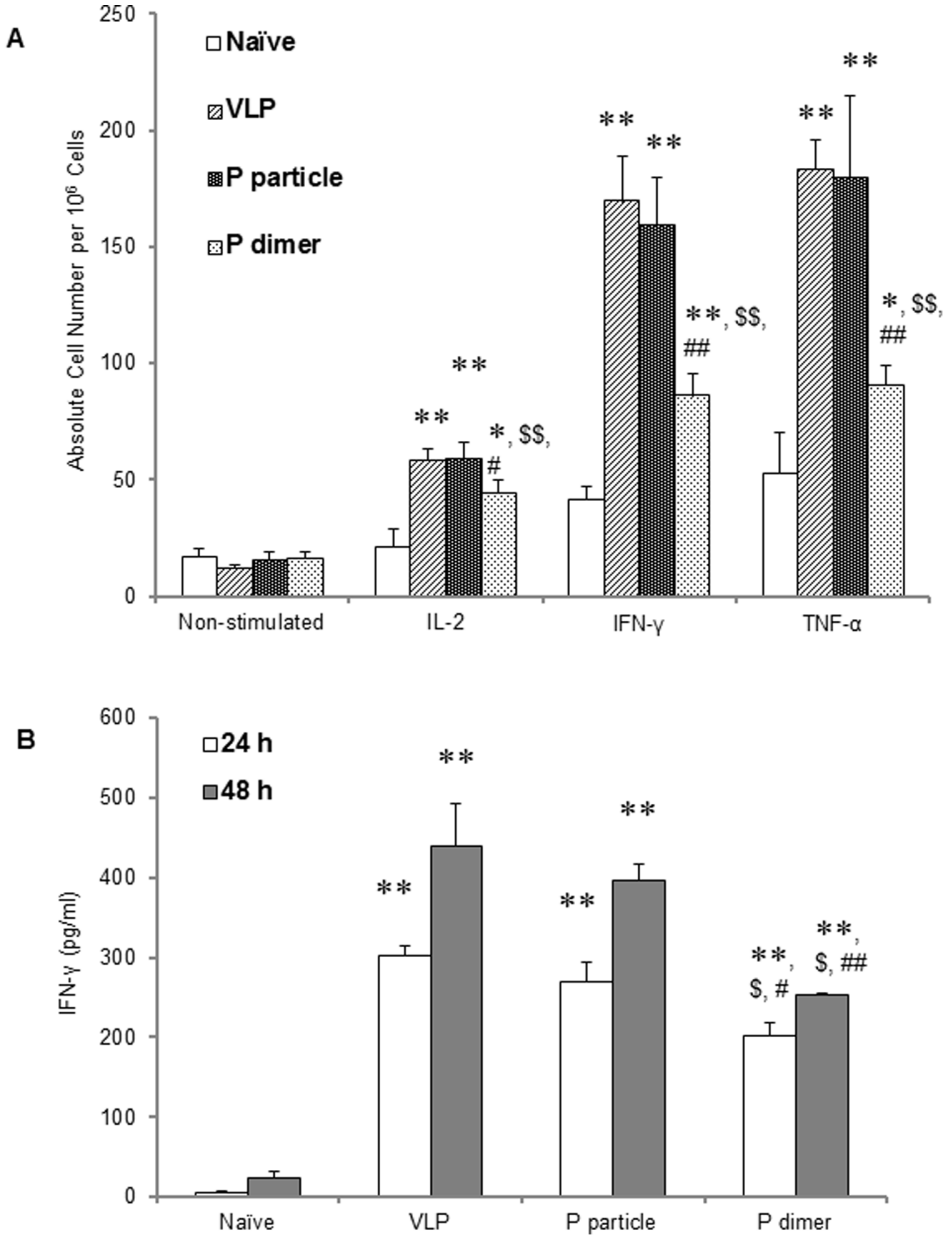
VA387-specific CD4^+^ T cells in immunized mice. (A) One week after the third immunization, splenocytes were collected and resuspended in RPMI-1640 with 3 µg/ml of brefeldin A and stimulated with 5 µg/ml of VA387 CD4 T cell epitope. Intracellular cytokines of IL-2, IFN-γ and TNF-α were stained as described Figure 4. (B) Splenocytes were collected from immunized mice and stimulated with VA387-specific CD4^+^ T cell epitope without brefeldin A. IFN-γ productions in culture supernatant 24 or 48 hours post stimulation were determined by ELISA. ∗ *P*<0.01 and ∗∗ *P*<0.001 were calculated vs. naïve mice control (Ctrl), while $ *P*<0.01 and $$ *P*<0.001 were calculated vs. VLP and # *P*<0.05 and ## *P*<0.01 vs. P particle, respectively. Experiments were repeated three times.

This was measured after an *in vitro* stimulation with VA387 CD4^+^ T cell epitope and intracellular cytokine staining. Compared with naïve control, splenocytes of all immunized mice produced significantly higher amount of IL-2, IFN-γ and TNF-α cytokines (Figure 5A, all *P*<0.01), in which P particle and VLP induced the highest antigen-specific cytokines, and thus specific CD4^+^ T cell responses, among all three immunogens (*P*<0.001). The P particle elicited similar amount of cytokines as VLP did and no significant difference was observed between the two immunogens for all three cytokines. Although P dimer could induce production of cytokines, their levels were significantly lower than those induced by VLP (*P*<0.01) and P particle (*P*<0.01). The IFN-γ in the supernatant of epitope-stimulated splenocytes in absence of brefeldin A were detected in mice of all three immunized groups at 24 and 48 hours post-stimulation, in which the IFN-γ in the VLP- and the P particle-immunized mice were significantly higher than that in the P dimer-immunized mice(Figure 5B, *P*<0.05). These results further suggest that, while both P domain complexes are able to induce NoV-specific CD4^+^ T cell response, the P particles induce a higher IFN-γ to a level comparable to that induced by the VLP.

### P domain complexes can induce maturation of bone marrow-derived dendritic cells (BMDCs)

**Figure 6:**
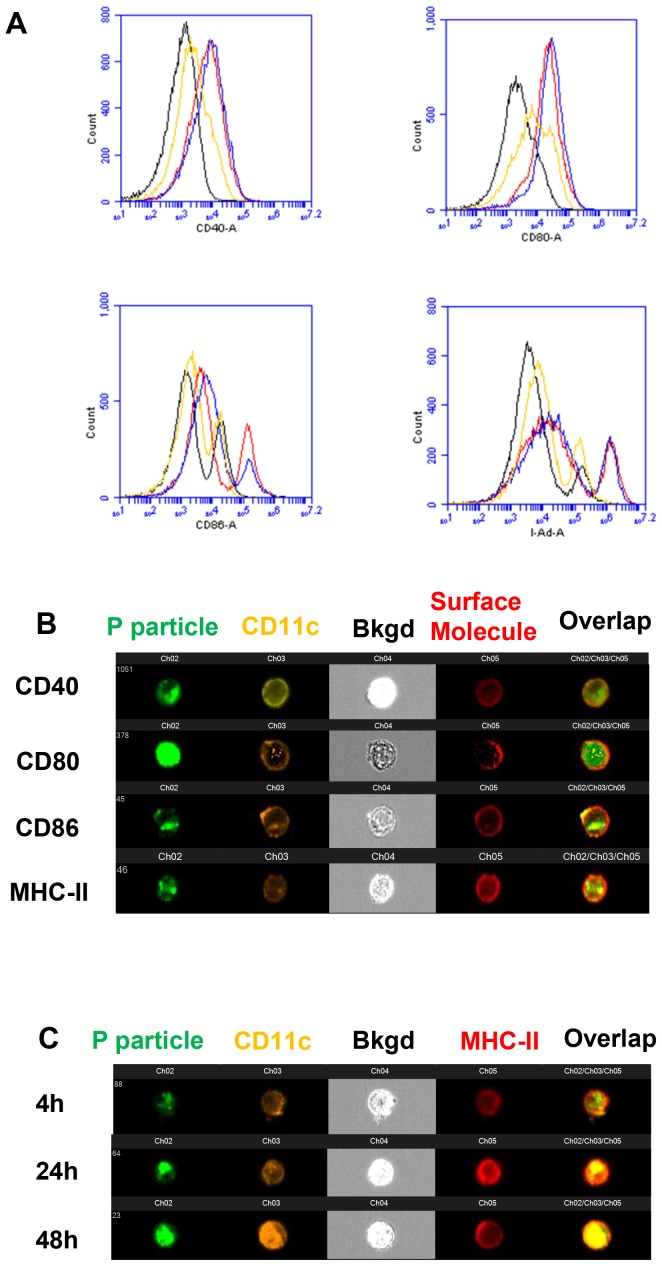
Dendritic cell (DC) maturation induced by norovirus P domain complexes. Bone marrow (BM)-derived DCs (BMDCs) were generated as previously described [34], [35]. BM cells were cultured with 20 ng/ml of GM-CSF and harvested on day 7. This protocol routinely yielded >90% CD11c^+^ cells as determined by flow cytometry. (A) For maturation assay, BMDCs were stimulated with 10 µg/ml of norovirus VLPs (red), P particles (blue), or P dimers (yellow) for 48 hours, respectively. BMDCs without stimulation (black) were used as control. Cells were harvested at 24 or 48 hours and stained with fluorochrome-conjugated CD11c together with CD40, CD80, CD86 and MHC-II for flowcytometric analysis. (B) ImagestreamX of co-stimulatory and MHC-II molecules were analyzed on BMDC stimulated with AF488-labeled-P particles at 24 hours post stimulation. (C) Kinetic of MHC-II molecule was analyzed on BMDC stimulated with AF488-labeled-P particles at 4, 24 or 48 hours post stimulation. Pictures are representatives of experiments which were repeated three times.

BMDCs from naive mice were studied to determine NoV antigen uptake and maturation of DCs. Significantly up-regulated CD40, CD80, and CD86, as well as MHC class II molecules on the surface of CD11c^+^ cells were observed 24 hours or 48 hours after stimulation (Fig. 6A, Table 1 and Table 2), indicating the maturation of DCs was induced by all three antigens. The antigen uptake occurred within 24 hour post stimulation of the antigens as demonstrated by ImageStreamX with of DCs using Alexa Fluro 488 (AF488)-labeled P particle (Figure 6B). The up-regulation of MHC-II molecules on CD11c^+^ cells seemed in parallel to the uptake of the P particle (Figure 6C). These data indicated that the internalization of the P particle up-regulated MHC-II molecules, which could facilitate the P particle antigen presentation from DCs to CD4^+^ T cells for the induction of adaptive immune responses. Similar scenario was also observed for the P dimer (data not shown).

**Table 1:** Dendritic cell maturation induced by norovirus VLPs, P particles and P dimers at 24 hours post stimulation.

	Naive	VLP	P particle	P dimer
CD40	2885.8±177.6	22046.8±1209.3 ^**^	20327.4±705.6^**, §^	4106.4±546.1 ^*, §§, ##^
CD80	12945.0±1374.0	38420.6±922.8 ^**^	34869.0±772.8 ^**, §§^	15594.6±810.3 ^**, §§, ##^
CD86	17642.4±1293.4	53666±682.4 ^**^	51719.4±1289.6 ^**, §^	22801.6±1601.1 ^**, §§, ##^
MHC-II	216839.2±13959.2	603645.2±13401.5 ^**^	571099.8±11078.7^**, §§^	357897.4±11174.3 ^**, §§, ##^

Bone marrow-derived dendritic cells (BMDCs) were stimulated with 10 µg/ml of norovirus VLPs, P particles, or P dimers for 24 hours. BMDCs without stimulation were used as control (Naïve). Cells were harvested and stained with fluorochrome-conjugated CD11c together with CD40, CD80, CD86 and MHC-II for flowcytometric analysis. Mean Fluorescence index (MFI) of each group was analyzed and shown in a format of average ± SD. * *P*<0.01 and ** *P*<0.001 were calculated vs. non-pulse control, while § *P*<0.05 and §§ *P*<0.01 were calculated vs. VLP, and ## *P*<0.001 vs. P particle, respectively. Experiments were repeated three times.

**Table 2:** Dendritic cell maturation induced by norovirus VLPs, P particles and P dimers at 48 hours post stimulation.

	Naive	VLP	P particle	P dimer
CD40	3304.4±140.7	24929.6±834.2^**^	22650.0±1097.2^**, §^	4987.8±219.7 ^**, §§, ##^
CD80	16031.4±1199.2	53012.4±2291.5 ^**^	48855.2±963.5^**, §§^	21753.0±1795.3 ^*, §§, ##^
CD86	16219.0±1368.4	58610.4±3052.6 ^**^	56053.2±3287.4 ^**^	21771.8±914.4 ^**, §§, ##^
MHC-II	220254.0±10417.3	685917.6±30537.0 ^**^	608873.4±14426.0^**, §§^	430893.2±6697.9 ^**, §§, ##^

Bone marrow-derived dendritic cells (BMDCs) were stimulated with 10 µg/ml of norovirus VLPs, P particles, or P dimers for 48 hours. BMDCs without stimulation were used as control (Naïve). Cells were harvested and stained with fluorochrome-conjugated CD11c together with CD40, CD80, CD86 and MHC-II for flowcytometric analysis. Mean Fluorescence index (MFI) of each group was analyzed and shown in a format of average ± SD. ** *P*<0.01 and ** *P*<0.001 were calculated vs. non-pulse controls, while § *P*<0.05 and §§ *P*<0.01 were calculated vs. VLP, and ## *P*<0.001 vs. P particle, respectively. Experiments were repeated three times.

**Figure 7:**
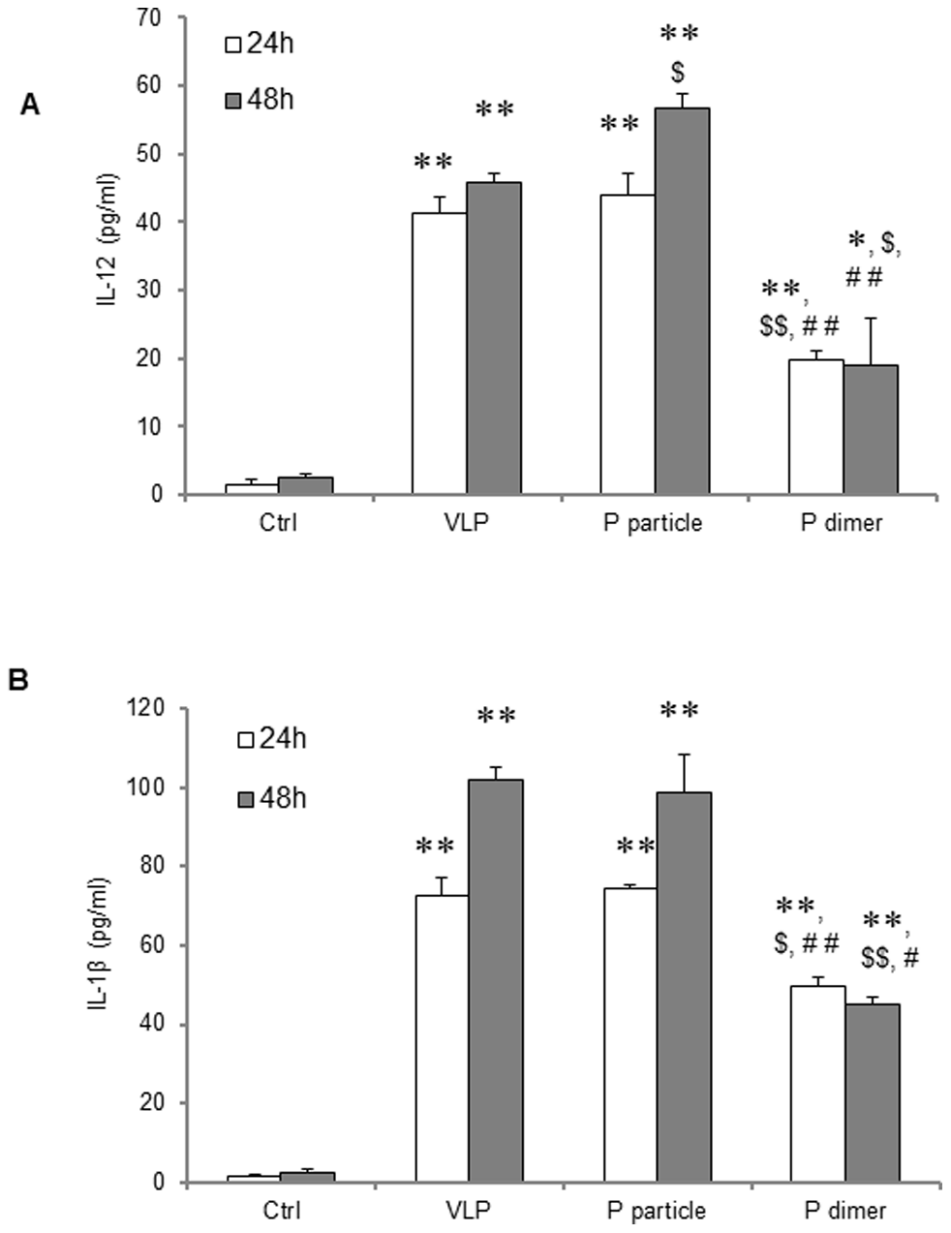
Cytokine production by mature bone marrow-derived dendritic cells (BMDCs). BMDCs were stimulated with 10 µg/ml of norovirus VLPs, P particles, or P dimers for 24 or 48 hours, respectively. BMDCs without stimulation were used as control (Ctrl). (A) IL-12 production in supernatant was determined by ELISA against IL-12 p70. (B) IL-1β production in supernatant was measured against IL-1β. ∗ *P*<0.05 and ∗∗ *P*<0.01 were calculated vs. non-stimulated control (Ctrl), while $ *P*<0.05 and $$ *P*<0.01 were calculated vs. VLP and # *P*<0.05 and ## *P*<0.01 vs. P particle, respectively. Experiments were repeated three times.

The P particle also stimulated high titer of IL-12, a Th1-polarized cytokine, similar as that stimulated by VLP in DC at 24 hours, or even higher than that of VLP at 48 hours (*P*<0.05) post stimulation (Figure 7A), while P dimer induced significantly lower amount of IL-12 (*P*<0.05 or *P*<0.01). Similar result was observed in the production of IL-1β, an important pro-inflammatory cytokine, at 24 and 48 hours post stimulation (Figure 7B, *both P*<0.01). The up-regulation of surface molecules and production of IL-12 as well IL-1β could not be inhibited by polymyxin B, indicating that these effects were not due to the lipopolysaccharide (LPS) contamination of the P domain complexes (data not shown).

### VA387-specific CD4^+^ T cell proliferation induced by antigen-pulsed DCs

**Figure 8:**
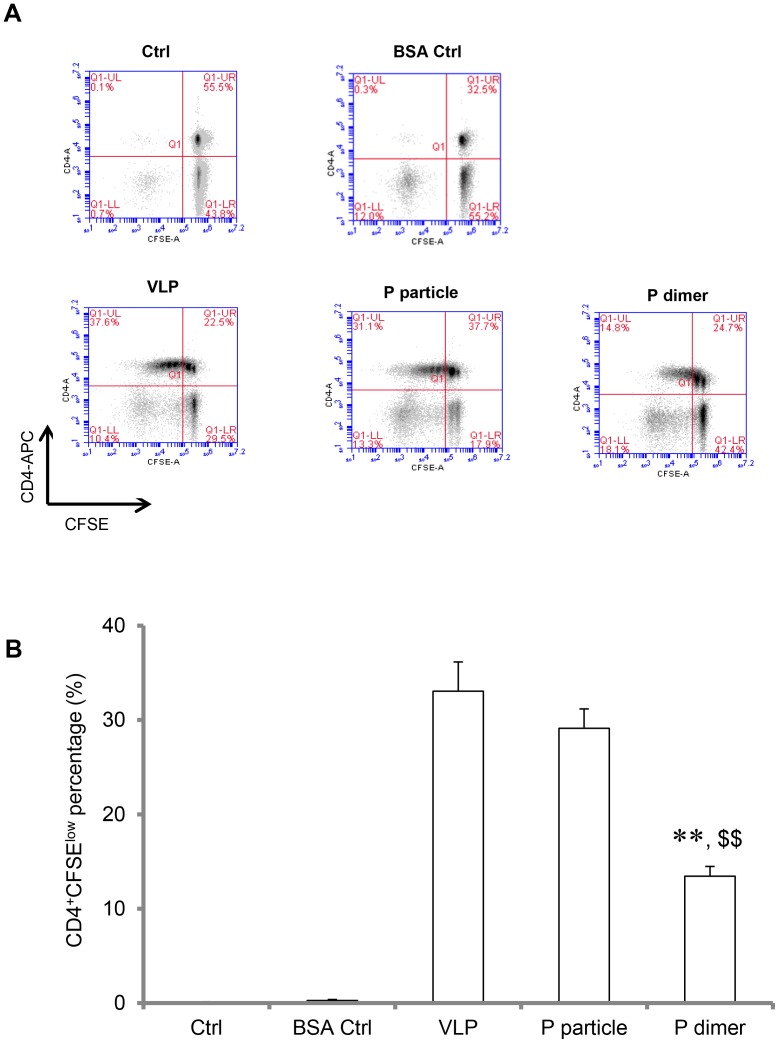
VA387-specific CD4+ T cells proliferation induced by antigen-pulsed dendritic cells (DCs). Splenocytes were isolated from immunized mice and labeled with carboxyfluorescein succinimidyl ester (CFSE). At the same time, mature bone marrow-derived DCs (BMDCs) pulsed with related antigens of VA387 VLPs, P particles, or P dimers were collected. Then, CFSE-labeled splenocytes were co-incubated with antigen-induced mature BMDCs for 5 days. Finally, cells were stained with APC-conjugated CD4 and were acquired by BD FACSCanto or BD Accuri™ C6. The resulting flow data were analyzed by FlowJo or Accuri™ C6 software. (A) Representative flow data of CD4^+^ T cell proliferation. (B) Percentages of proliferating CD4^+^ T cells (CD4^+^CFSE^low^ population) were shown in bar graph. ∗ *P*<0.001 and ∗∗ *P*<0.0001 were calculated vs. VLP, while $ *P*<0.001 and $$ *P*<0.0001 were calculated vs. P particle. Experiments were repeated three times.

T cell proliferation assay was performed to examine if the P particles-induced mature DCs can elicit antigen-specific T cell response. Both P particle- and VLP-pulsed DCs induced strong CD4^+^ T cell proliferation at 31.3% and 37.6%, respectively (shown as CD4^+^CFSE^low^ population, *P*>0.05) (Figure 8A and B), indicating that VA387 antigen-specific CD4^+^ T cells generated by immunization of the two antigens profoundly proliferated. However, the P dimer only induced 14.8% proliferation, which is significantly lower than that induced by the P particle or VLP (*P*<0.001, Figure 8A and B). As expected, T cell proliferation was barely found in naïve DCs or BSA-pulsed DCs (Figure 8A and B). These results suggested that VA387 P particle immunization generated specific VA387 CD4^+^ T cells, which would efficiently proliferate when encountered VA387 or similar NoVs presented by antigen-presenting cells.

## Discussion

Due to the lack of an efficient animal model for human NoVs, little is known about the immune responses of NoV infection. We showed in this study that NoV VLP and two P domain complexes, the P particles and P dimers, are efficiently presented by dendritic cells (DCs) for eliciting cellular immunity. This conclusion is supported by the significant induction or activation of several cellular immunity factors by the three antigens, including : 1) central memory CD4^+^ T cell phenotypes (CD4^+^ CD44^+^ CD62L^+^ CCR7^+^), 2) polyclonal CD4^+^ T cells, 3) an antigen-specific CD4^+^ T cell response, 4) bone marrow-derived dendritic cell (BMDC) maturation, and 5) mature dendritic cells (DCs) to elicit proliferation of specific CD4^+^ T cells targeting VA387 P domain. These results provide strong supports on the application of these recombinant viral capsid antigens as vaccines against NoVs, since human NoVs remain uncultivable *in vitro*.

The application of NoV VLPs as a candidate vaccine against NoVs has been studied extensively [23], [29], [37]–[39] and a phase II clinical trial has been performed, which showed a promising result of protection against NoV infection and illness [23]. The application of NoV P particles as a candidate vaccine has also been proposed recently based on their easy production and high immunogenicity [27], [30], [40] and authentic HBGA receptor binding properties in comparison with VLPs. The general similarity and difference between the immune systems of human and mouse are known and mice are frequently used to test vaccine candidates before they are used for human trials. In case of human noroviruses, available data have shown that the immunogenicity of VLP vaccines are similar in mouse and human and the VLP-induced serum antibodies from both human and mouse are able to block the binding of norovirus to HBGA receptors [17], [27], [30]. However, since NoV P domain constitutes only a half of the viral capsid protein and the P domain complexes are smaller in sizes, a question has been raised on their potential lower immunogenicity compared with VLPs. This concern now can be addressed by the comparable high responses of both humoral and cellular immunity by both VLPs and P particles. In our recent study we also proposed to apply the P particle as a vaccine platform to present different foreign antigens for vaccine development. The observed high humoral and cellular immunity induced by the P particle backbone may also help to explain the reported immune enhancement of the presented antigens.

The high cellular immunity induced by the P domain complexes indicated that NoV P domain possesses T cell epitopes, which was also supported by previous findings of others. For example, utilizing overlapping peptide libraries, LoBue *et al* [41] identified CD4^+^ T epitope in the P1 domain of GII.4 Farmington Hills strain, a highly conserved region within the GII.4 genotype. In our study we predicted CD4^+^ T cell epitope (FYQEAAPAQSDVAL) for BALB/c mice based on the T cell epitope prediction tools by the *Immune Epitope Database (IEDB) Analysis Resource* (website: http://tools.immuneepitope.org/main/). This predicted T cell epitope turns out to be highly effective, which is consistent with those in previous published literatures [41], [42]. As expected, this epitope stimulated the cytokine responses in P complex-immunized mice, indicating the establishment of T cell immune responses against NoV P domain.

In this study we also noticed that the immunogenicity of the recombinant P dimers is significantly lower than that of VLPs or P particles for both humoral and cellular immunities, which is consistent with the observations in our previous studies focusing on antibody responses [5], [31]. The lower immunogenicity of the P dimers may be due to its smaller size (69 kDa) and lower valence of the antigenic structures compared with that of VLPs (>1 mDa, 180mer) and P particles (830 kDa, 24mer). We also noticed that the immunogenicity of P particles and VLPs under the same dosages (30 µg/mouse) is similar for both humoral and cellular immunities. These data suggested that the size and valences of P particle is properly sufficient for a a highly efficient immunogen or vaccine, which support the proposal of the P particle as a vaccine against NoVs in consideration of its easier production in *E. coli* than VLPs that require a eukaryotic expression system.

In a previous study Tamminen *et al* reported that NoV P particles induced significantly weaker antibody and CD4^+^ T cell responses than those of VLPs [43]. However, questions have been raised on the efficiency of P particle formation of their reagent [31], which may be the major reason of the observed weaker immune responses of their P particles, as the immunogenicity of the P dimers is significantly lower than that of the P particles [[27] and this report]. In addition, different immunization schemes could also result in different outcomes. Tamminen *et al* immunized mice intramuscularly or intradermally with a low dose (10 µg/mouse) for only two times, while we used a dose of 30 µg/mouse intranasally for three times. A direct comparison using the same doses, dosages and immunization routes may be the only way to address the discrepancies.

DCs are antigen-presenting cells to initiate host immune responses, in which DCs capture pathogens and present their antigens to adaptive immune cells, such as T cells [44]. We demonstrated in this study that DCs captured and presented the NoV antigens of capsid protein or P domain to T cells to elicit adaptive immune responses, although the detailed mechanism remains unknown. It has been shown that DCs can internalize exogenous antigens through different manners, including receptor-mediated endocytosis [45], phagocytosis [46] or macropinocytosis [47]. Although further clarification is required, our preliminary data appear to exclude phagocytosis and macropinocytosis because specific inhibitors did not influence the maturation of DCs induced by NoV P particle (data not shown). Moreover, the exogenous antigens need to be presented by DCs not only to MHC-II molecules for CD4^+^ T cell responses, but also to MHC-I molecules for cytotoxic CD8^+^ T cell responses. Further studies focusing on how DCs process NoV P particle would help us to understand and improve the usefulness of this candidate vaccine and vaccine platform.
