# Correction: Norovirus P Particle Efficiently Elicits Innate, Humoral and Cellular Immunity

**DOI:** 10.1371/annotation/01406b4d-7869-4e7f-b294-4330ab641e85

**Published:** 2014-01-02

**Authors:** Hao Fang, Ming Tan, Ming Xia, Leyi Wang, Xi Jiang

An error occurred in the first sentence of the "Reagents" section in the Materials and Methods: "anti-I-A^b^ (AF6-120.1)" is incorrect and should instead read "anti-I-A^d^ (39-10-8)."

